# Glycine Transporter 1 Inhibitors: Predictions on Their Possible Mechanisms in the Development of Opioid Analgesic Tolerance

**DOI:** 10.3390/biomedicines12020421

**Published:** 2024-02-12

**Authors:** Anna Rita Galambos, Zsolt Tamás Papp, Imre Boldizsár, Ferenc Zádor, László Köles, Laszlo G. Harsing, Mahmoud Al-Khrasani

**Affiliations:** 1 Department of Pharmacology and Pharmacotherapy, Faculty of Medicine, Semmelweis University, Nagyvá-rad tér 4, H-1445 Budapest, Hungary; galambos.anna@phd.semmelweis.hu (A.R.G.); papp.zsolt@med.semmelweis-univ.hu (Z.T.P.); boldizsar.imre@stud.semmelweis.hu (I.B.); zador.ferenc@pharma.semmelweis-univ.hu (F.Z.); 2 Department of Oral Biology, Semmelweis University, H-1089 Budapest, Hungary; koles.laszlo@dent.semmelweis-univ.hu

**Keywords:** opioid analgesic tolerance, glycine transporter 1, N-methyl-D-aspartate receptors, µ-opioid receptors

## Abstract

The development of opioid tolerance in patients on long-term opioid analgesic treatment is an unsolved matter in clinical practice thus far. Dose escalation is required to restore analgesic efficacy, but at the price of side effects. Intensive research is ongoing to elucidate the underlying mechanisms of opioid analgesic tolerance in the hope of maintaining opioid analgesic efficacy. N-Methyl-D-aspartate receptor (NMDAR) antagonists have shown promising effects regarding opioid analgesic tolerance; however, their use is limited by side effects (memory dysfunction). Nevertheless, the GluN2B receptor remains a future target for the discovery of drugs to restore opioid efficacy. Mechanistically, the long-term activation of µ-opioid receptors (MORs) initiates receptor phosphorylation, which triggers β-arrestin-MAPKs and NOS-GC-PKG pathway activation, which ultimately ends with GluN2B receptor overactivation and glutamate release. The presence of glutamate and glycine as co-agonists is a prerequisite for GluN2B receptor activation. The extrasynaptic localization of the GluN2B receptor means it is influenced by the glycine level, which is regulated by astrocytic glycine transporter 1 (GlyT1). Enhanced astrocytic glycine release by reverse transporter mechanisms as a consequence of high glutamate levels or unconventional MOR activation on astrocytes could further activate the GluN2B receptor. GlyT1 inhibitors might inhibit this condition, thereby reducing opioid tolerance.

## 1. Introduction

Looking for safe analgesics and adjuncts and repurposing existing medications to treat different pain entities are still challenges in pain research. The medicinal arsenal for treating mild to severe forms of acute pain includes non-steroidal anti-inflammatory drugs (NSAIDs), minor analgesics, such as paracetamol, and opioid agonists. In this regard, the general guide known as the WHO ladder, which can assist clinicians in treating cancer pain, includes these analgesics; however, opioid types are indispensable medications in this ladder, specifically when the intensity and frequency of pain is increased and cannot be adequately controlled by NSAIDs or other minor analgesics. Indeed, there is no debate on the effectiveness of opioid analgesics in relation to the management of cancer pain or noncancer chronic pain at the initiation of therapy. In the short-term treatment of pain, adverse effects related to the gastrointestinal tract develop, but generally, they are treatable with antiemetics, laxatives, and other drugs [1]). However, with long-term opioid analgesic treatment, the development of analgesic tolerance creates an obstacle, and to overcome it, dose escalation is required. Undoubtedly, further dose escalation results in benefits and risks for patients. Opioid tolerance occurs after long and repeated treatment with opioid agonists and is defined as a decrease in opioid potency, which is reflected by an increase in ED_50_ or EC_50_ values. These values are indicative of a pharmacological shift to the right in the dose/concentration–response curves of test opioid agonists, such as morphine [2,3]. In noncancer chronic pain, opioid analgesic tolerance does also occur, and the consequence of opioid dose escalation is that the patient is exposed to the risk of adverse events, including overdose and opioid use disorders (OUD) as well as other opioid side effects, as mentioned above [4,5,6]. Many preclinical and clinical studies have been conducted to understand the underlying mechanisms of opioid analgesic tolerance. In fact, µ-opioid receptors (MORs) mediate the analgesic effect of the most used opioid agonists in clinical practice. These indices indicate that opioid analgesic tolerance mediated through MORs is a clinical drawback hindering long-term opioid treatment. Furthermore, pharmacological blockade of spinal MORs with opioid antagonists, such as naloxone or H-D-Phe-Cys-Tyr-D-Trp-Arg-Thr-Pen-Thr-NH2 (CTAP) (a highly selective MOR antagonist), abolishes the analgesic effect of systemic morphine and fentanyl [7]. This means that elucidating spinal mechanisms that contribute to opioid analgesic tolerance could provide new knowledge that would lead to developing novel drug classes or at least adjuvants to taper off opioid doses. Indeed, there are many proposed mechanisms of opioid analgesic tolerance development, but none of them have brought significant value in the clinical setting. However, the diverse mechanisms related to opioid analgesic tolerance offer future research avenues that might ultimately unlock the solution to opioid tolerance. At the signal transduction level, MORs cause desensitization and downregulation (see Table 1), yet compensatory/opponent processes, such as neuroadaptations, occurred upon long-term exposure to opioid analgesics, such as morphine [3,8]. These processes encompass consequences related to opioid analgesic efficacy and homeostatic adaptation changes (opposite effect e.g., hyperalgesia). In this regard, the dose escalation effect is not only limited to the above-mentioned side effects, including opioid analgesic tolerance, but also can precipitate or exacerbate opioid-induced hyperalgesia [3]. The most promising achievements related to counteracting opioid analgesic tolerance was the application of competitive and non-competitive antagonists of ionotropic N-methyl-D-aspartate acid glutamate receptors (NMDARs), namely, MK-801 (dizocilpine), ketamine, and phencyclidine (PCP). In this regard, a large amount of evidence on the contribution of these receptors to opioid analgesic tolerance has been published in recent decades [9]. Alongside these studies, additional papers have also focused on the impact of spinal glutamate receptors in opioid analgesic tolerance, particularly with NMDARs, which are located on the pre- and postsynaptic membrane [10]. Furthermore, there are studies describing compounds that inhibit opioid analgesic tolerance by blocking GluN2A-2D receptor-operated ion channels, such as MK-801 [11,12]. However, these inhibitors bear several side effects, including cognitive impairment and dissociative behaviors [13,14]. Drugs devoid of these side effects that directly or indirectly affect the function of GluN2A-2D-receptor-operated ion channels in relation to opioid analgesic tolerance would be worth investigating in the future.

In this review, we shed light on undiscovered targets in opioid analgesic tolerance, namely glycine transporter type 1 (GlyT1). The prediction is supported by the following evidence from the literature: (i) GlyT1 regulates extrasynaptic glycine concentrations; (ii) glycine acts as a co-agonist at GluN2A-2DRs; (iii) GluN2B is predominantly found in the extrasynaptic region; and (iv) GluN2BR plays a crucial role in the development of opioid analgesic tolerance.

## 2. N-Methyl-D-aspartate Acid Glutamate Receptors as Key Players in the Development of Opioid Analgesic Tolerance

As mentioned in the introduction, in recent decades, extensive research has been conducted to elucidate the underlying mechanisms of opioid analgesics tolerance at different pharmacodynamic levels. At the cellular level, several postulated mechanisms have been proposed for opioid analgesic tolerance, as described in Table 1, though the exact mechanisms responsible are poorly understood. With respect to receptor desensitization and opioid analgesic tolerance development, several mechanisms have been proposed, including one that relies on the activation of NMDARs [23,24,25]. This mechanism is supported by the fact that MK-801, a highly potent and selective noncompetitive NMDAR antagonist, attenuates the development of morphine analgesic tolerance in mice [12,26]. Further preclinical studies have shown that opioid tolerance that developed upon long-term treatment with intrathecal morphine is attenuated by MK-801 or 5-AP, competitive NMDAR antagonist, or GM1 ganglioside (an intracellular PKC inhibitor) in rats [27,28]. All these data shed light on the involvement of glutamate receptors in the development of opioid analgesic tolerance. The next section focuses on deciphering the receptors that mediate the glutamate effects related to opioid analgesic tolerance, particularly in the spinal cord.

### 2.1. Metabotropic Glutamate and NMDA-Type Glutamate Receptors

Glutamate receptors are classified as metabotropic glutamate receptors (mGluRs) [29,30] (Table 2) and ionotropic glutamate receptors (iGluRs) [31]. They belong to the G-protein coupled receptor and tetrameric ionotropic receptor superfamilies, respectively. Based on the activator ligands, iGluRs are further classified as NMDA (Table 3), AMPA (α-amino-3-hydroxy-5-methyl-4-isoxazoleproprionic acid), and kainate receptors [32]. Owing to the signaling pathway feature, metabotropic receptors mediate slower responses when compared to ionotropic receptors [33]. The mGluRs include eight subtypes, named mGluR1 through mGluR8 [34]. Furthermore, based on the homology of amino acid sequences, agonist selectivity, and their interactions with intracellular second messengers, mGluRs are further classified into three groups, namely groups I, II, and III. The group I receptors belong to G protein-coupled receptors, namely Gaq/11, and mediate their effects through inositol phosphate 3/Ca^2+^ (PLC) signal transduction. Likewise, groups II and III also belong to G protein-coupled receptors, but they couple to Gi/o type G-proteins, and the signal transduction mechanism relies on the inhibition of adenylate cyclase. Almost all mGluRs, except mGluR6, show a ubiquitous expression pattern in the CNS both in neurons and glial cells, though some subtypes have specific regional expression, as described in Table 2. The group I (mGluR1 and mGluR5), group II (mGluR2 and mGluR3), and group III (mGlu4,6,7, and 8) receptors are activated by 3,5-dihydroxyphenylglycin (3,5-DHPG), LY354740, and L-2-amino-4-phosphonobutyrate (L-AP4), respectively [34,35]. The group II receptors (mGluR3-LI) have been reported to show a pattern of distribution in the brain and spinal cord areas related to pain processing. In this regard, in the brain stem and spinal cord, the distribution was weak, whereas in the principle sensory trigeminal nucleus, spinal trigeminal nuclei, paratrigeminal nucleus, and spinal cord dorsal horn, the distribution was moderate or strong [35,36]. With respect to pain control related to group I mGluRs, several preclinical studies have shown that the activation of these receptors results in pronociceptive action in different animal pain models [37,38,39,40]. Since the objective of the present work was to map the contributions of GlyT1 to the development of opioid analgesic tolerance, extrasynaptically localized NMDARs are reviewed in the next section.

### 2.2. Glutamatergic Ionotropic NMDA Receptors and Opioid Analgesic Tolerance

NMDARs belong to the ligand-gated ion channel family and mediate glutamate neurochemical transmission, as described in the previous section. These receptors have a tetrameric structure, and each subtype is composed of different subunits, including GluN1, GluN2A, GluN2B, GluN2C, GluN2D, GluN3A, and GluN3B, as shown in Table 3. NMDARs’ macromolecular structures are built up from four subunits designated as GluN1 and GluN2A-D. These heterotetrameric structures host two copies of GluN1 (obligatory) and two copies of one of the GluN2s (GluN2A, 2B, 2C, or 2D) [31]. Thus, the NMDA-operated ion channels exhibit different characteristics that result in functional diversity [53]. The two GluN2 subunits (an orthosteric binding site) bind glutamate, and the two GluN1 subunits (co-agonist binding sites) bind glycine or D-serine (Table 4). An example for such diversity is a tetramer of 2GluN1-2GluN2B (heterotetramer).

The opening of the NMDA receptor-operated channel is unique and complex. It requires the simultaneous occupation of the agonist binding site by glutamate and the co-agonist binding sites either by glycine or D-serine, along with the depolarization of cells to remove Mg^2+^ blockade. NMDA-operated ion channels are non-ion selective; in addition to Ca^2+^, they allow for the entry of monovalent cations, such as Na^+^ and K^+^, into the cells. It has been proposed that the co-agonist binding site of NMDAR is equivalent to the second agonist binding site but was altered during evolution. The pattern distribution and the subclasses of glutamatergic ionotropic NMDARs are presented in Table 3. Several data suggest that GluN2B receptors are predominantly localized extrasynaptically; see Table 3 and Traynelis et al. [54]. 

Overactive GluN2B receptors are thought to play a key role in analgesic tolerance elicited by the repeated administration of opioid agonists [55]. On the contrary, different pharmacological interventions, which decrease NMDAR overactivity, inhibit the development of opioid tolerance in analgesia. Shimoyama and coworkers [56] reported that the NMDAR channel blockers ketamine and MK-801 can suspend the analgesic tolerance of opioids. These data are in line with that above mentioned (see Section 2) and reviewed in the literature [57,58,59,60,61,62]. It has also been reported that the negative allosteric modulators of the GluN2B receptor, such as ifenprodil or Ro25-6981, reduced NMDAR activity and suspended the development of opioid tolerance in nociception [63].

**Table 3:** Distribution of N-methyl-D-aspartate acid glutamate receptor family in central nervous system areas related to pain processing.

Glutamate Receptor Subunits	Area (Region)	Distribution Pattern	Subject	References
GluN1 (NMDAR1)	GluN1-2: widely distributed in the CNS GluN1-1: in rostral regions (e.g., cortex) GluN1-4: in caudal regions (e.g., brainstem) Dorsal horn of spinal cord Spinal cord Spinal cord: laminae I–III	+++ +++ +++ +++ +++ +++	Rat	[64,65,66,67,68]
GluN2A (NMDAR2A)	Cerebral cortex Substantia gelatinosa neurons (synaptic localization) Spinal cord	+++ +++	Rat	[67,69,70]
GluN2B (NMDAR2B)	Telencephalon and thalamus DRG neurons (primary afferent neurons) Substantia gelatinosa neurons (extrasynaptic localization) Spinal cord: laminae I–III Spinal cord: lamina II (and IX)	+++ +++ * ++/+++ +/++	Rat	[67,68,69,70,71]
GluN2C (NMDAR2C)	All regions except cerebellar cortex Spinal cord: laminae I–III Spinal cord	+ 0 0	Rat	[67,68,69]
GluN2D (NMDAR2D)	Brainstem, cortex Substantia gelatinosa neurons Spinal cord	+ * 0	Rat	[67,69,70]
GluN3A (NR3A)	Thalamus (VPL) Cervical spinal cord: in proximity to the dorsal horn	++ +++	Rat (postnatal day 16)	[72]
GluN3B (NR3B)	Cerebral cortex Spinal cord: laminae I–II (and VIII-IX) (**) Spinal cord: laminae III–VI	+++ ++/+++	Rat	[73]

^+^ The degree of distribution pattern; * electrophysiological study; ** in the dorsal horn, parvalbumin-positive interneurons are NR3B-positive (these neurons are glycinergic interneurons). IUPHAR terminology used, with previously used nomenclature in brackets.

**Table 4:** Endogenous and exogenous modulators for N-methyl-D-aspartate acid glutamate receptors [31].

Glutamate Re-ceptor Subunits	Endogenous Agonists		Exogenous Agonists		Exogenous Antagonists		Channel Blocker
	Glutamate Site	Glycine Site	Glutamate Site	Glycine Site	Glutamate Site	Glycine Site	
GluN1 (NMDAR1)	L and D-Asp	Glycine D-serine	NMDA HQA *	(+)-HA966 *	-	5,7-DCKA	
GluN2A (NMDAR2A)	L and D-Asp	Glycine D-serine	NMDA HQA *	(+)-HA966 *	D-AP5	5,7-DCKA	Mg^2+^, MK-801, ketamine, phencyclidine, amantadine.
GluN2B (NMDAR2B)	L and D-Asp	Glycine D-serine	NMDA HQA *	(+)-HA966 *	D-AP5	5,7-DCKA	Mg^2+^, MK-801, ketamine, Phencyclidine, amantadine.
GluN2C (NMDAR2C)	L and D-Asp	Glycine D-serine	NMDA HQA *	-	D-AP5	5,7-DCKA	Phencyclidine, ketamine, amantadine, Mg^2+^, MK-801.
GluN2D (NMDAR2D)	L and D-Asp	Glycine D-serine	NMDA HQA *	-	D-AP5	5,7-DCKA	Mg^2+^, MK-801, amantadine, ketamine, phencyclidine.
GluN3A (NR3A)							
GluN3B (NR3B)							

* Electrophysiological study; aspartic acid (Asp); homoquinolinic acid (HQA); 5,7-Dichlorokynurenic acid (DCKA).

## 3. Glycine Transporter Type 1 and N-Methyl-D-aspartate Acid Glutamate Receptors: Their Locations and Functions in the Glial–Neural Tripartite Synapse

We have hypothesized interactions among GlyT1, NMDA, and opioid receptors in the development of opioid analgesic tolerance. The morphological basis for this interaction is the tripartite model, which consists of an astrocytic process, a presynaptic axon terminal, and a postsynaptic element with the synaptic cleft [74]. It has been demonstrated that MORs are present on glutamatergic axon terminals and astrocytes [75]. NMDARs are expressed in both pre- and postsynaptic neurons, and GlyT1 is localized in astrocytes and in glutamatergic axon terminals interacting with NMDA-type glutamate receptors [76]. However, these receptors and transporters are present in the glial–neural tripartite synapse in various forms.

Typical and atypical MORs are coupled either to the conventional G_i_ signal transduc-tion or, in the unconventional form, to G_q_ protein. These receptors are operated with cAMP and PKA phosphorylation or DAG/IP_3_ and PKC-mediated phosphorylation. In controlling analgesia, these MORs operate in glutamatergic axon terminals or astroglia cells, respectively [75] 

GluN2B receptors are predominantly localized extrasynaptically. This localization creates possibilities for glutamates diffused out from neighboring synaptic clefts or astrocytes to influence the function of GluN2B receptors [77]. This pattern of neuronal organization occurs in the spinal cord, cortex, and thalamus [78]. On the other hand, GluN2A receptors have been suggested to be synaptically expressed. The prerequisite for the activation of the formerly mentioned receptors is the simultaneous presence of the following three components: glutamate, co-agonists (either glycine or D-serine), and the removal of Mg^2+^ from NMDA receptor ion channels by depolarization.

Thirdly, GlyT1 can primarily be detected with glial fibrillary acidic protein (GFAP) in astroglia cells and has a role in building up extrasynaptic glycine concentrations by bi-directional operation [79,80]. GlyT1 consists of 12 transmembrane (TM) domains. TM1-5 and TM6-10 form a gate for 2Na^+^ and 1Cl^−^ ion transport [81]. In addition to glial GlyT1, this transporter was also reported to be present in the vicinity of GluN2B receptors (GluN2BRs) in the membranes of postsynaptic neurons. Neural GlyT1 was suggested to assure the co-agonist glycine for this receptor activation [82]. To understand the mechanisms and the function of spinal glycine homeostasis, the next section sheds light on ligands affecting GlyTs.

### 3.1. Classification of Glycine Transporter Inhibitors

As we attempt to build a hypothesis on how the tripartite model is involved in the development of opioid analgesic tolerance, it is essential to review spinal glycine homeostasis, particularly in the vicinity of GluN2BRs. This means that GluN2BRs, GlyTs, and MORs would contribute to the development of opioid analgesic tolerance. Nevertheless, GlyT1 would be of interest because it plays a fundamental role in the regulation of extrasynaptic glycine levels [24,83,84]. In this regard, we summarize the various GlyT1 and GlyT2 inhibitors to help the readers understand the entire spinal glycine regulatory system in the hope of making successful predictions about the development of opioid analgesic tolerance. 

Several GlyT inhibitors have been described in the last two decades. Although they selectively inhibit either astrocytic GlyT1 or neuronal GlyT2, animal models of human disorders have also pointed to the need for non-selective GlyT inhibitors [85]. Modeling neuropathic pain in rats has indicated the involvement of both types of GlyTs in the pathological alterations of spinal cord neuronal circuitries [85,86,87,88]. 

At present, GlyT inhibitors can be classified based on their chemical structures. The very first GlyT1 inhibitor that was discovered was glycyldodecylamide (GDA), which exhibited modest inhibitory potency [89]. This compound called attention to the importance of membrane lipids around GlyT1 in the binding of ligands to the transporter molecule. The amide-head in GDA suggests a possible ionic bonding interaction of this compound with Tyr128 in the transporter [90]. Compounds derived from the endogenous glycine uptake inhibitor arachidonic acid also indicate the importance of the lipid tail, which may interact with the lipid environment of the cell membrane. In addition to the lipophilic part, oleyl-L-carnitine also contains a basic head group [83]. Despite some similarities in the chemical structures of GDA and oleyl-L-carnitine, the former acts as an inhibitor of GlyT1, and the latter has been shown to more likely block GlyT2 activity.

While the role of the basic head group in the binding of inhibitors to GlyTs has been shown, other GlyT inhibitors contain free carboxylic groups derived from glycine or sarcosine. Sarcosine is a modest but selective GlyT1 inhibitor, with a possible binding site in the cavities formed by the Tyr128-Tyr302-Ser303-Leu304-Gly305 amino acids within the transporter molecule [90]. Although sarcosine is considered to be a competitive GlyT1 antagonist [91], we found it to exhibit a substrate-type inhibition of the transporter, shifting its operation to the reverse mode [90]. Lipid-containing GlyT2 inhibitors with a free carboxylic group instead of a basic head have also been reported (N-arachidonyl-glycine, N-oleyl-glycine) [92].

Based on the selective GlyT1 inhibitory property of sarcosine, a great number of GlyT1 inhibitors were synthesized by attaching extended lipophilic aryl groups to the N atom [93,94]. These compounds are called sarcosine-based GlyT1 inhibitors, which exhibit a competitive or non-competitive type of transporter inhibition [79,95]. Examples of these inhibitors are NFPS, Org-24461, SSR-130800, LY-2365109, and SzV-1997. Compounds from Merck and Allelix (ALX1393) containing glycine instead of sarcosine have also been synthesized and possess inhibitory effects on GlyT1 and GlyT2, respectively [96].

**Figure 1:**
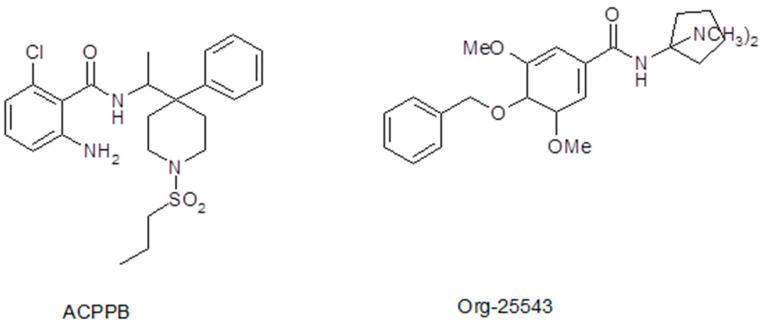
Chemical structures of benzamide derivatives GlyT1 inhibitor ACPPB and GlyT2 inhibitor Org-25543. We speculate that these two compounds may be sources for synthesizing a novel non-selective GlyT1/GlyT2 inhibitor.

Because of the adverse effects of sarcosine-based GlyT1 inhibitors (often due to their pharmacokinetic properties), chemical synthesis has turned toward non-sarcosine-based GlyT1 inhibitors. A great number of compounds have also been synthesized in this series, and some of them have undergone clinical investigations as well. Of these compounds, we found the benzamide derivatives particularly interesting. Compounds containing the benzamide skeleton exhibited selective GlyT1 (ACPPB, SSR-504734, GSK-1018921) or GlyT2 (Org-25543) inhibitory properties (Figure 1). Therefore, we believe that consideration of the chemical structures of ACPPB and Org-25543 may be the basis for identifying non-selective GlyT inhibitors with a novel pharmacological profile in different experimental conditions or even in clinical use. Nevertheless, the development of selective GlyT1 inhibitors seems to be ideal for therapeutic purposes in the context of the present review, namely for opioid analgesic tolerance.

### 3.2. Compounds Acting as Ligands for Glycine Transporter Type 1 and NMDA Receptor Interactions: Some Operational Characteristics

Glycine acts as an agonist on glycine receptors (strychnine-sensitive binding site) and as a co-agonist on the strychnine-insensitive glycine binding site of GluN2A-D type NMDARs (Table 4) in the spinal cord. Glycine is also the substrate for GlyTs, and it is released from astrocytes or glycinergic nerve endings in the CNS.

**Figure 2:**
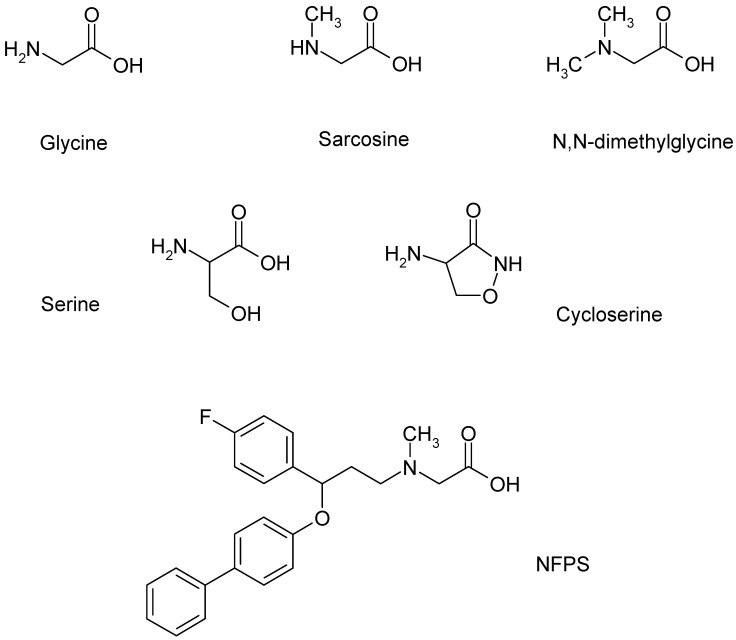
Ligands of GlyT1 and NMDA receptors, which form a functional interaction in the glia cell, presynaptic axon terminal, and postsynaptic neuron tripartite model. NFPS is a sarcosine-based GlyT1 inhibitor.

**Figure 3:**
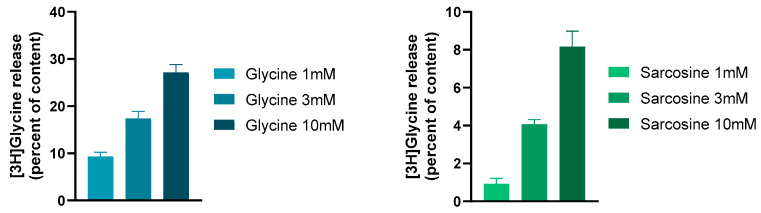
The GlyT1 ligands glycine and sarcosine evoked a concentration-dependent and external Ca^2+^-independent release of [^3^H]glycine from rat hippocampal slices. This release was due to the reverse-mode operation of GlyT1. The slices were incubated with [^3^H]glycine, and then superfused with aerated (95% O_2_/5% CO_2_) and preheated (37 °C) Krebs bicarbonate buffer. Twenty-two 3 min fractions were collected. Drugs were added to the hippocampal slices from fraction 5 and maintained throughout the experiments. At the end of superfusion, the tissue content of radioactivity and the radioactivity released from the tissues were determined. The GlyT1 non-competitive antagonist NFPS (0.1 mM) reversed the effect of 3 mM sarcosine on [^3^H]glycine release: it was 0.68 ± 0.21 and 4.07 ± 0.24 per cent of the content released in the presence and absence of NFPS, respectively. Student’s t statistics for two-means, *p* < 0.001, mean ± S.E.M., n = 4–8. Data were taken and redrawn with permission [90].

Other compounds may also bind as co-agonists to the glycine binding sites of NMDARs or act as substrates for GlyT1 (Figure 2). The structural similarities between glycine and sarcosine raise the possibility that sarcosine has a co-agonist role in NMDARs [91]. As discussed above, sarcosine was one of the very first GlyT1 inhibitors to be found. This inhibition of GlyT1 may be the substrate type, eliciting [^3^H]glycine efflux from glia cells by shifting GlyT1 operation into the reverse mode [90]. Moreover, this effect of sarcosine, which is identical to that of the natural substrate glycine, was suspended by the addition of the GlyT1 inhibitor NFPS (Figure 3, legend).

**Figure 4:**
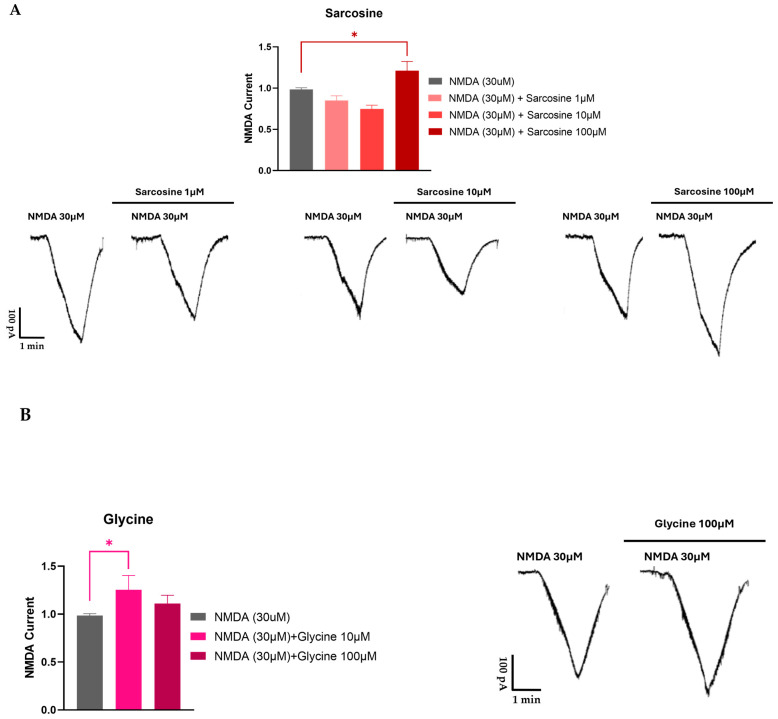
Electrophysiological recording from rat prefrontal cortex slices for sarcosine (**A**) and glycine (**B**). Sarcosine induced a biphasic effect on NMDA receptor-mediated currents. Ten-day-old Wistar rat pups were decapitated, and coronal slices containing the medial prefrontal cortex (PFC) were prepared from the brain using a vibrotome. The slices were stored in a holding chamber before use. A single slice was transferred into a recording chamber and superfused (2.5–3 mL/min) with aerated artificial cerebrospinal fluid at room temperature. Whole cell patch clamp recordings were implemented. Pyramidal cells in the PFC were visualized with an upright microscope. Borosilicate glass patch pipettes were filled with standard intracellular solution; the tip resistance was 5–7 MΩ. The membrane currents were recorded in the voltage–clamp configuration of the amplifier at a holding potential of −70 mV. The data were filtered at 2 kHz with a lowpass filter of the amplifier, digitized at 5 kHz, and stored in a computer. NMDA (30 μM) was applied three times (T1, T2, and T3) for 1.5 min with 10 min intervals. Test drugs were added to the bath 5 min before and during the third application of NMDA. Since the amplitudes showed great variabilities among the cells, the effects at T3 were presented as the T3/T2 ratio. The T3/T2 ratios were summarized as the mean ± SEM of n. Statistical significance was established by one-way ANOVA, followed by Dunnett’s post hoc test. *p* < 0.05 was considered to be statistically significant (*).

Sarcosine, in addition to its action on GlyT1, also acts directly on NMDARs as a co-agonist [91]. Using a whole cell patch clamp recording in rat prefrontal cortex slices, we found that sarcosine exhibited a bidirectional effect. At lower concentrations, it inhibited the NMDA receptor-mediated currents, whereas, at higher concentrations, it enhanced them (see Figure 4). Moreover, these experiments also suggested a possible partial agonist effect of sarcosine on NMDA receptor-mediated currents. These findings are contradictory to those of McBain et al. [97], who reported that sarcosine did not bind to the glycine-binding site of NMDARs expressed in Xenopus oocytes. Nevertheless, Lee and colleagues [98] showed that sarcosine, in addition to its action as a GlyT1 inhibitor, also potentiated NMDAR function by acting as a co-agonist at the glycine binding site of NMDARs. Sarcosine alone does not initiate a response but enhances NMDA receptor-mediated currents when applied with glutamate [98]. What is interesting in our results is that at lower concentrations (1 and 10 μM), sarcosine reduced NMDA receptor-mediated currents (Figure 4). At present, we can explain the observed effect as follows: (i) The measured NMDA receptor-mediated currents are the result of the binding of both glutamate and glycine (present in tissue); (ii) at a low concentration, sarcosine behaves as a partial agonist on glycine binding sites, and consequently, hinders the physiological effect of glycine; (iii) at high concentrations, sarcosine inhibits GlyT1, and as a result, more glycine is available in the vicinity of NMDARs. On the other hand, it is clear that glycine is a full agonist and shows an effect on one direction (Figure 4). However, the presented data for glycine reveal a decreased effect of glycine at higher concentrations. This feature might be related to the ability of glycine to evoke NMDAR desensitization. This finding might correlate with that of Zhang and coworkers [91], who concluded that NMDAR desensitization is more likely to occur with glycine than with sarcosine.

The sarcosine analogue N,N-dimethylglycine, which did not affect GlyT1 operation in our [^3^H]glycine efflux experiments, also binds to the NMDA receptor glycine binding site (Table 5). The potencies of sarcosine and N,N-dimethylglycine in the brain slices were found to be equal. N,N-Dimethylglycine acts more as a partial agonist at the glycine binding site of NMDA receptors [98].

Another glycine analog, N-ethylglycine, reduced GlyT1-dependent glycine uptake, acting as an alternative substrate for GlyT1. It is a selective and competitive inhibitor of GlyT1 without altering GlyT2 activity [99].

D-Serine acts as a co-agonist on synaptic NMDA receptors, with a subunit composition of GluN1/GluN2A receptors [100]. D-Cycloserine was reported to be a partial agonist at the glycine binding site of NMDA receptors [101].

## 4. Interplay between Glycine Transporter Type 1 and N-Methyl-D-aspartate Acid Glutamate Receptors in Reversal of Opioid Tolerance: A Hypothesis

**Figure 5:**
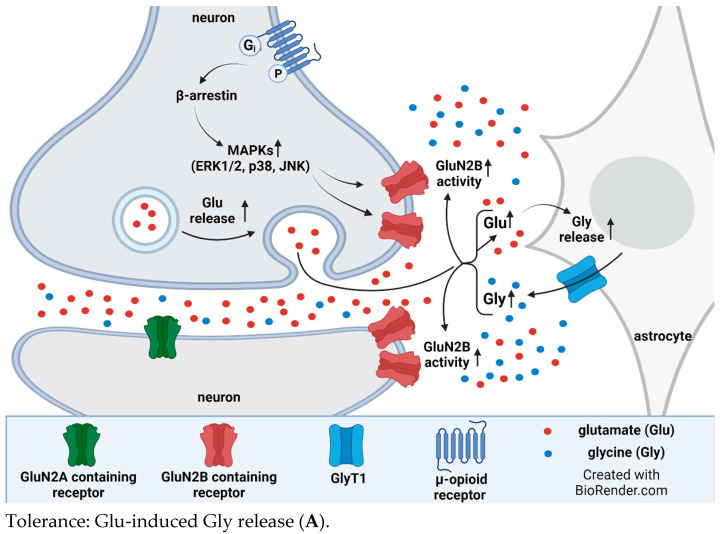

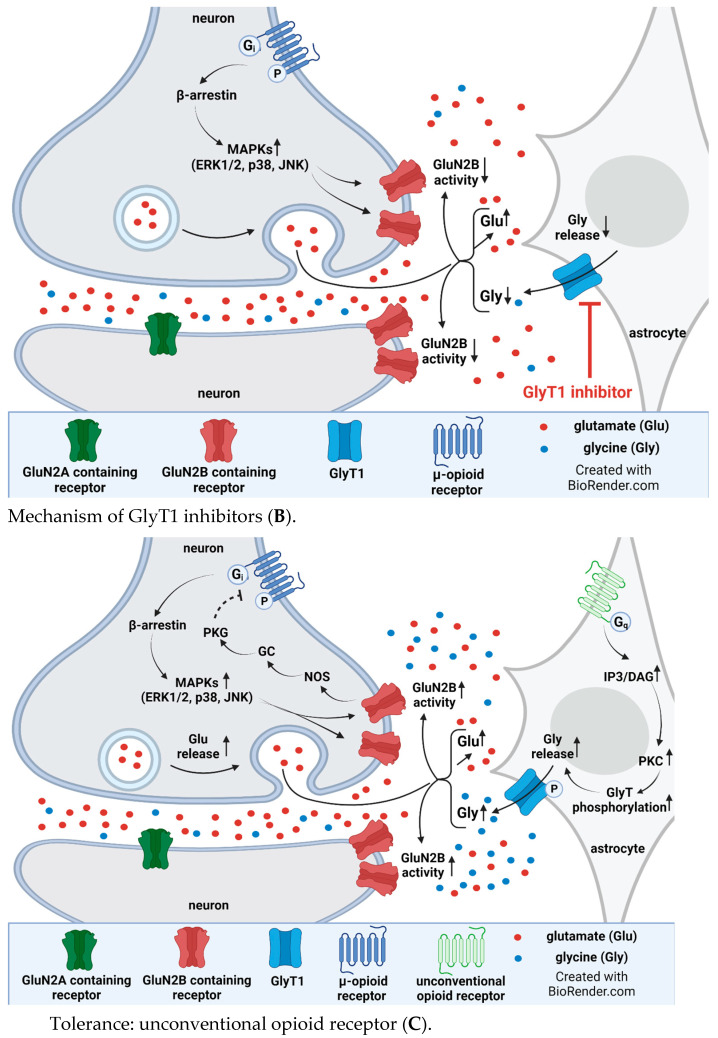

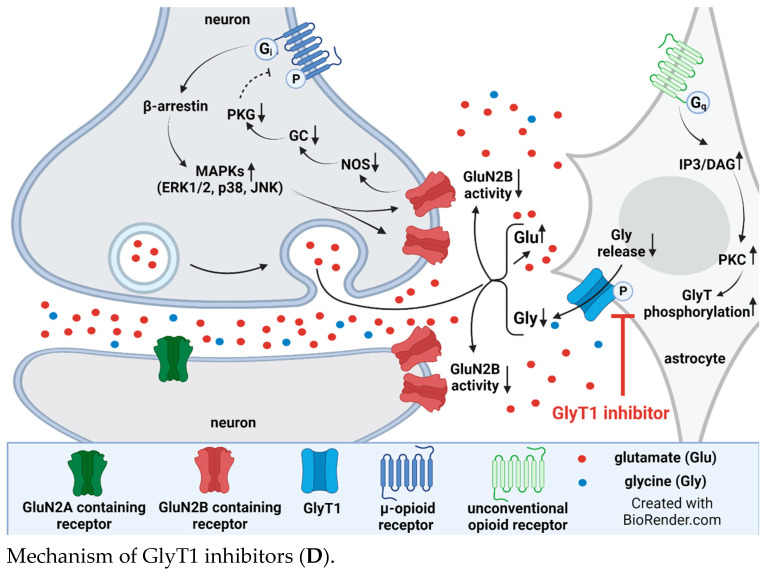
(**A**,**B**) Chronic treatment with opioid analgesics, such as morphine, initiates MOR-mediated MAPK activation through the phosphorylation process that occurs in the presynaptic nerve ending. This activation encompasses the extracellular signal-regulated kinases 1 and 2 (ERK1/2), c-Jun amino-terminal kinases, and p38. As a result, the GluN2A and GluN2B receptors are activated. NMDAR activation in this condition enhances glutamate release. The activation of GluN2A and GluN2B receptors requires glutamate and co-agonist (glycine). The source of glutamate is the activated neuron, whereas the source of glycine is either glycinergic neurons or astrocytes [16,23]. The illustrations were created by BioRender, with agreement numbers 2023 IS268LVHRP for 5/A and 2023 JT268LWY14 for 5/B. (**C**,**D**) A glial–neuronal tripartite model of opioid tolerance and its potential reversal by GlyT1 inhibitors. G_q_ protein-coupled unconventional MORs are activated by acute or repeated morphine administration in astroglia cells. The increase in IP_3_/DAG production and [Ca^2+^]i triggers PKC-mediated phosphorylation, which shifts the balance of the uptake-release mode of GlyT1 toward release-mode operation. The consequent high extracellular glycine levels upregulate extracellular NMDA GluN2B receptors, which inhibit the signal transduction of µ opioid receptors resulting in the development of opioid tolerance. GlyT1 inhibitors inhibit the bidirectional operation of the transporter and decrease elevated extracellular glycine levels, reversing NMDA receptors’ sensitivity. This adapts opioid receptor sensitivity to normal levels. Abbreviations: NOS, nitric oxide synthases; GC, guanylyl cyclase; PKG, protein kinase G. The illustrations were created by BioRender, agreement numbers 2023 IU268LVTVC for 5/C and 2023 EO268LVZVX for 5/D. Figure 5 was adapted from previous works [75,109,114].

To the best of our knowledge, the interaction between opioid and glycine systems in relation to opioid analgesic tolerance has not been elucidated thus far. Sufficient results are available for MOR-mediated analgesia and possible mechanisms contributing to the development of opioid analgesic tolerance, as mentioned in the introduction. With respect to analgesia, morphine reduces pain reactions by the activation of conventional MORs in the dorsal horn of the spinal cord [7], and the inhibition of these receptors by intrathecal MOR antagonists has been reported. Also, opioid agonists decrease glutamate release from glutamatergic nerve endings, and this effect may be at least part of the evoked analgesia [102]. MORs’ conventional signal transduction following opioid treatment and the consequent development of opioid tolerance encompasses mitogen-activated protein kinase (MAPKs: ERK1/2, p30, JNK) activation through β-arrestin. These kinases activate presynaptic GluN2B receptor [23]; see Figure 5.

However, the administration of morphine also activates unconventional opioid receptors expressed in astroglia cells [75,103]. These receptors are coupled to Gq proteins, and their signaling evokes an increase in the protein kinase C (PKC)-mediated phosphorylation of GlyT1. It has been shown that phorbol 12-myristate 13-acetate, a PKC activator, evokes the inhibition of [^3^H]glycine uptake in HEK 293 and C6 glioma cells [104,105]. The inhibition of glycine uptake by phosphorylated GlyT1 may be a similar mechanism that has been reported for other plasma membrane neurotransmitter transporters [106]. Along this line, we speculate that the PKC-mediated phosphorylation of GlyT1 inhibits the uptake-mode operation, which may be associated with an increased rate of the reverse-mode operation of the transporter [107]. The shift in the bidirectional operation of GlyT1 to the release mode evoked by PKC-mediated phosphorylation may lead to a marked increase in extracellular glycine concentrations, similar to a number of other experimental conditions [108]. An increase in extracellular glycine concentrations results in a co-agonist-induced overactivation of extracellular GluN2B receptors and strengthens the inhibition of opioid receptors, which then causes the development of opioid tolerance in analgesia. This negative influence exerted by NMDA receptors on opioid receptors occurs at the signal transduction pathways of the two receptors (NOS-guanylyl cyclase-PKG signaling) [109].

We have found that GlyT1 inhibitors block transporter operation in both directions in several experimental conditions [108]. The inhibition of the reverse-mode operation of GlyT1 leads to a decrease in extracellular glycine concentrations, reducing the co-agonist activation of the extracellular GluN2B receptor, which then results in the suspension of the negative influence on MOR activity. In a condition like this, the sensitivity of MORs to morphine or other opioid agonists may be restored, and opioid tolerance development may be delayed (Figure 5).

To further strengthen our hypothesis about a concomitant activation of MOR and the inhibition of GlyT1 as a mechanism responsible for delaying the development of opioid analgesic tolerance, in vivo studies are required to support this hypothesis.

Another possible mechanism is that long-term treatment with opioid analgesic agonists, such as morphine, causes an increase in glutamate levels in the spinal cord, as described previously [110]. An increase in glutamate levels can trigger glycine release, which in turn, together with increased glutamate, can enhance GluN2B receptor activity. This condition can give rise to the development of opioid analgesic tolerance (Figure 5A–D). The excitatory amino acid transporter (EAAT)-mediated release of D-aspartate, a drug that mimics glutamate, was lowered in the presence of GlyT1 inhibitors in synaptosomes of the spinal cord and hippocampus [111,112]. The result may be a decrease in GluN2B receptor activation, which has been described to largely be involved in opioid analgesic tolerance [113].

Our hypotheses focus primarily on GlyT1 operation in the development of opioid analgesic tolerance. It is also important to determine which brain areas are primarily favored for the interactions among GlyT1, NMDA-, and opioid receptors. It would also be interesting to see whether a hypothesis on the regulatory role of GlyT1 operation in opioid tolerance is valid only in analgesic tolerance or tolerance for other opioid effects also. Further elucidation of these questions is currently the prime interest of our laboratory.

## 5. Discussion and Conclusions

Overactive GluN2B receptors are thought to play a key role in analgesic tolerance elicited by the repeated administration of opioid analgesics. In fact, different pharmacological interventions, which decrease NMDAR overactivity, inhibit the development of opioid tolerance in analgesia. Thus, the NMDAR channel blockers ketamine and MK-801 and the negative allosteric modulators of the GluN2B receptor (ifenprodil, Ro25-6981) reduce NMDAR activity and suspend the development of opioid tolerance in nociception. Along this line, here, we suggest a potential interaction between GlyT1 and GluN2B receptors and conventional and unconventional MORs in the development of opioid analgesic tolerance. The morphological basis for these interactions is the presynaptic axon terminal, postsynaptic element with the synaptic cleft, and the astrocytic processes. These contributors, namely GlyT1 and NMDARs, are present in different forms in this tripartite model. Indeed, the functional interaction between GlyT1 and NMDARs is now generally accepted [115]. This interaction is based on the fact that extrasynaptic glycine concentrations, which are regulated by GlyT1, determine NMDAR activity by modulating their co-agonist sites. GlyT1 and NMDAR interaction may be either stimulatory or inhibitory in different CNS pathologies. In this regard, in schizophrenia, NMDARs are believed to be hypoactive; the NMDAR channel blockers PCP and ketamine worsen patients’ conditions, and GlyT1 inhibitors were developed in the hope of restoring the NMDAR hypofunction observed in this disorder [116,117,118,119,120,121]. On the contrary, NMDAR hyperfunction has been reported in depression, hypoxic/anoxic conditions, and convulsions [119,122,123,124,125]. The NMDAR channel blocker ketamine suspends NMDAR overactivity in these conditions [126,127,128], and GlyT1 inhibition exerts antidepressant, neuroprotective, and anticonvulsive effects [122,129,130]. In addition, ketamine and dizocilpine suspended opioid analgesic tolerance, suggesting NMDAR hyperfunctionality in repeated opioid receptor stimulation [56]. Owing to these literature data, we hypothesize that GlyT1 inhibitors could delay opioid analgesic tolerance development. Thus, depending on pathological conditions, NMDAR channel inhibitors and GlyT1 inhibitors may influence NMDAR activity in the identical or opposite directions. Since GlyT1 regulates the extracellular glycine concentration, reduced NMDAR activity in schizophrenia may be the result of overactive GlyT1 operation in the uptake mode. On the other hand, enforced release-mode operation of GlyT1 leads to elevated extracellular glycine concentrations causing NMDAR hyperactivity in depression, neurodegeneration, seizures, or opioid analgesic tolerance. Thus, GlyT1 inhibitors may either increase or decrease extrasynaptic glycine levels, and this optimization may increase hypofunctional and decrease hyperfunctional NMDAR operation in various CNS disorders. Therefore, we suggest that GlyT1 inhibitors possess a more complex operation than just glycine uptake inhibition.

To the best of our knowledge, neither preclinical nor clinical studies have been carried out so far to elucidate the impact of GlyT1 inhibitors on the development of opioid analgesic tolerance. Large evidence exists regarding the efficacy of GlyT1 inhibitors in experimental schizophrenia models, though they have failed in phase III clinical studies. As mentioned above, the key player is NMDAR, which undergoes a hypofunctioning state in schizophrenia or hyperfunctioning state in opioid analgesic tolerance. As a limitation, glycine concentrations and their impact on spinal NMDARs to delay opioid analgesic tolerance necessitate the examination of GlyT1 inhibitors following acute and chronic administration. Yet, the safety of these drugs needs to be assessed under these protocols focusing on organ functions, particularly respiration and motor operation.

## Figures and Tables

**Table 1:** The main enzymes, proteins, and mechanisms involved in opioid analgesic tolerance.

Enzyme/Protein/Mechanism	Role in Opioid Tolerance	References
β-arrestin 1 and 2	Receptor desensitization Receptor trafficking	[15,16]
GRK2, 3, 4, and 5	Receptor desensitization and trafficking via receptor phosphorylation	[15,16,17,18]
PKCα, γ, and ε	Receptor desensitization and trafficking via receptor phosphorylation	[18,19,20,21]
CAMKII	Receptor desensitization via receptor phosphorylation	[16,18]
ERK1/2	Receptor desensitization via arrestins	[16]
JNK	Receptor desensitization	[16,21]
Dynamin	Receptor trafficking	[16,22]
Rybophorin I (glycosylation)	Receptor trafficking	[18]
PATs (palmitoylation)	Receptor trafficking	[18]
Ubiquitination (participating proteins not described)	Receptor trafficking	[18]

Abbreviations: CAMKII, calcium–calmodulin kinase II; ERK1/2, extracellular signal-regulated kinase 1/2; GRK, G protein receptor kinase; JNK, c-Jun N-terminal kinase; PAT, protein acyltransferase; PKC, protein kinase.

**Table 2:** Distribution of metabotropic glutamate receptors in central nervous system areas related to pain processing.

Receptor Group	Receptor Subtype	Area (Region)	Distribution Pattern	Subject	Connection to Pain Sensation	References
Group I	mGluR1	DRG	6.8%	Rat, mouse, human, monkey	↑ Nociceptive behaviors Inflammatory pain Neuropathy	[41,42,43,44,45,46]
		PA				
		DH				
		VH	90%			
		Thalamus				
		PFC				
	mGluR5	PA		Mouse, rat, human, monkey		[42,43,44,45,46,47,48,49]
		DH				
		Thalamus	90%			
		PAG				
		PFC				
Group II	mGluR2	DRG	51.6%	Rat, human	Activation induces analgesia in inflammatory and neuropathic pain	[41,43,46]
		Thalamus				
		PFC				
	mGluR3	DRG				[41,42,43,44,46]
		DH				
		Thalamus				
		PFC				
Group III	mGluR4	DH		Rat, human	Anti-hyperalgesic effect of mGlu group III activation	[42,43,44,46]
		Thalamus				
		VH				
		PFC				
	mGluR6	Superior colliculus, hypothalamus, olfactory bulb		Mouse, rat		[50]
	mGluR7	DH	90%	Rat, human		[42,43,44,46]
		VH				
		Thalamus				
		PFC				
	mGluR8	DRG	75–80%	Rat, human		[41,42,43,46,51,52]
		DH				
		Thalamus				
		PAG				
		PFC				
		Peripheral axons	40%			

Abbreviations: PFC, prefrontal cortex; DH, dorsal horn; VH, ventral horn; DRG, dorsal root ganglia; PA, primer afferent; PAG, periaqueductal grey.

**Table 5:** Ligands for glycine transporter type 1 and N-methyl-D-aspartate acid glutamate receptors.

Ligands	GlyT1	NMDA Receptors
Glycine	Substrate	Co-agonist
Sarcosine	Substrate inhibitor Competitive antagonist	Co-agonist
N.N-Dimethylglycine	Ineffective	Partial agonist
N-Ethylglycine	Competitive inhibitor	
D-Serine	-	Co-agonist
D-Cycloserine	-	Partial agonist
NFPS	Competitive inhibitor Non-competitive inhibitor	No effect

References: [90,91,94,98,99,100,101].
